# Clinical Spectrum of Drug-Induced Movement Disorders: A Study of 97 Patients

**DOI:** 10.5334/tohm.554

**Published:** 2020-10-26

**Authors:** Anjali Chouksey, Sanjay Pandey

**Affiliations:** 1 Department of Neurology, Govind Ballabh Pant Postgraduate Institute of Medical Education and Research, New Delhi, IN

**Keywords:** Drug-induced movement disorder, Tardive syndromes, dopamine receptor blocker agents

## Abstract

**Background:**

Drug-induced movement disorders (DIMDs) are commonly encountered, but an often-under-reported subgroup of movement disorders.

**Objectives:**

We aimed to highlight the spectrum of DIMDs in patients taking different groups of drugs at our movement disorder center.

**Methods:**

It is a cross-sectional descriptive study including 97 consecutive DIMDs patients diagnosed over the past two years (2017–2019).

**Results:**

The mean ± standard deviation (SD) age of our study population was 35.89 ± 17.8 years (Range-2–80 years). There were 51 males and 46 females. Different DIMDs observed included tardive dystonia (n = 41; 42.2%), postural tremor (n = 38; 39.2%), parkinsonism (n = 32; 33%), tardive dyskinesia (n = 21; 21.6%), acute dystonia (n = 10; 10.3%), neuroleptic malignant syndrome (NMS) (n = 2; 2.1%), and others [(n = 10; 10.30%) including chorea and stereotypy each in 3; acute dyskinesia in 2; and myoclonic jerks and acute akathisia each in 1 patient]. Of these 97 patients, 49 had more than one type of DIMDs while 48 had a single type of DIMDs. In our study 37 (38%) patients had received non-dopamine receptor blocking agents (non-DRBA), 30 (31%) patients had received dopamine receptor blocking agents (DRBA), and 30 (31%) patients had received both DRBA and non-DRBA.

**Conclusions:**

Tardive dystonia was the most common DIMDs observed in our study. Our DIMDs patients were younger than other reported studies. We observed a significant number of non-DRBA drugs causing DIMD in our study as compared to previous studies. Drug-induced parkinsonism (DIP) was the most common DIMDs in the DRBA group. Tardive dystonia was the most common DIMDs seen in DRBA + non-DRBA group and the second most common in the DRBA and non-DRBA group. The postural tremor was the most common DIMDs in the non-DRBA group.

## 1. Introduction

Drug-induced movement disorders (DIMDs) constitute an important treatable subgroup of movement disorder. Recently, different DIMDs are classified into eight categories, according to the fifth edition of the ‘Diagnostic and Statistical Manual of Mental Disorders (DSM-5)’ [1]. Dopamine receptor blockers agents (DRBAs) like neuroleptics, antiemetics, and gastrointestinal promotility agents, are the most common drug group causing movement disorder as a side effect. Apart from DRBAs, DIMDs have also been reported after various non-DRBAs such as selective serotonin reuptake inhibitors (SSRIs), opioids, phenytoin, gabapentin, and anaesthetic agents like propofol, fentanyl, sevoflurane, and morphine [2,3,4]. Most of the studies on DIMDs are done by psychiatrists on the patients with underlying psychiatric illness who are on neuroleptics. In contrast, very few studies have been done by neurologists [5]. Consequently, the focus of most of the published studies is on antipsychotic-induced movement disorders. The current knowledge of the DIMDs caused by other groups of drugs is primarily based on isolated case reports and small case series. So there is a poor reflection of different DIMDs caused by the broad range of drugs encountered in general neurology and movement disorder clinics in the existing literature. We aimed to characterize the spectrum of DIMDs and its associated risk factors, and find out the frequency of various DRBA and non-DRBA drugs implicated in causing DIMDs.

## 2. Methods

### 2.1. Data collection and inclusion criteria

The present study is a cross-sectional descriptive study of 97 consecutive DIMD patients of all age groups evaluated in our movement disorder clinic and indoor services at our tertiary care centre over the past two years (2017–2019). The study was approved by our institutional ethics committee. The demographic details of the patients and the information regarding the onset and duration of abnormal movement, primary illness, details of the treatment taken and the duration of drug intake were obtained from the patient and caregivers and also reviewed from the previous medical records when available. The Naranjo score was calculated to estimate the likelihood of a causal relationship between a drug and an adverse abnormal movement for each patient based on the information provided. The score ranges are classified as follows: scores of ≥9 indicate a definitive adverse drug reactions (ADRs), scores from 5 to 8 a probable ADRs, scores between 1 and 4 a possible ADRs, and 0 is considered a doubtful ADRs. Only drugs with a score equal to or more than 5 were included [6]. We excluded the patients who had i) levodopa-induced dyskinesia ii) presence of known causes of secondary movement disorder and iii) appearance of DIMDs after one year of stopping the drug. All patients underwent detailed clinical examination, and video documentation after appropriate informed written consent. The diagnosis of DIMDs was confirmed, and abnormal movement was classified into eight different categories as defined by DSM-5 criteria for DIMDs [2]. We extended suspected etiologic drugs in the DSM-5 criteria to include non-DRBAs as used in previous studies [7,8]. Depending on the drugs taken by the patients, we divided them into three groups namely DRBA only, non-DRBA only and non-DRBA + DRBA.

### 2.2. Statistical analysis

The collected data was entered in MS-EXCEL, and statistical analysis was done using the SPSS-PC-17 version. Qualitative data were analyzed using descriptive analysis which involved various measures of central tendency, measures of dispersion, and frequency counts. Tests of statistical significance were applied based on the data obtained. A chi-square test was used for the categorical values, and the t-test was used for numerical values. P-value < 0.05 was considered to be significant.

## 3. Results

### 3.1. Demographics of the study population

The mean age of our study population was 35.89 ± 17.8 years (Range-2–80 years). The patients in three drug groups did not differ in terms of their age (Table 1). There were 51 males and 46 females. Different aetiologies for which the suspected drugs were prescribed included psychiatric disorders (n = 49, 50.5%), seizure disorder (n = 20, 20.6%), gastrointestinal disorder (n = 13,13.4%), migraine (n = 6, 6.2%), seizure disorder with psychiatric disorders (n = 2, 2.1%) and others (n = 7, 7.2%). A statistically significant positive correlation coefficient was observed between age and tardive dyskinesia (r = 0.203, P < 0.05), meaning that the likelihood of tardive dyskinesia increased as age increased (Table 2). There was a trend towards positive correlation between age and DIP but it was not statistically significant (r = 0.189, P = 0.058).

**Table 1:** Comparison of different groups of drugs causing drug-induced movement disorder.

Characteristics	DRBA (N = 30)	Non-DRBA (N = 37)	DRBA + Non-DRBA (N = 30)	p-value

Mean age (in years) ± SD	34 ± 18	28 ± 18	33.7 ± 16.8	.501
Frequency of different DIMD^#				
DIP	16 (53.3%)	5 (13.5%)	11(36.7%)	**.0017**
Tardive dystonia	11 (36.6%)	13 (35.1%)	17 (56.7%)	.156
Tardive dyskinesia	10 (33.3%)	7 (18.9%)	4 (13.3%)	.150
Postural tremors	5 (16.6%)	20 (54%)	12 (40%)	**.007**
Acute dystonia	6 (18.2%)	3 (8.1%)	1 (3.3%)	.089
NMS	1 (3%)	0	1 (3.3%)	–
Others*	5 (16.6%)	4 (10.8%)	1 (3.3%)	–

^ DIMDs are classified as per DSM-5 criteria. # Total ≠ 100%, as some patients had more than one type of DIMD. * Others include chorea (n = 3), acute dyskinesia (n = 2), stereotypy (n = 3), myoclonic jerks (n = 1) and acute akathisia (n = 1). Abbreviations: DIMD – drug-induced movement disorders, DIP – drug induced parkinsonism, DRBA – dopamine receptor blocking agents, NMS – neuroleptic malignant syndrome.

**Table 2:** Age distribution of different drug induced movement disorders.

Age (In years)	Tardive dystonia N = 41	Postural Tremor N = 38	DIP N = 32	Tardive dyskinesia N = 21	Acute dystonia N = 10	Others* N = 10	NMS N = 2

0–<10	0	0	1	1	0	0	0
10–<20	7	3	2	4	3	3	0
20–<30	12	16	7	2	2	2	0
30–<40	6	10	6	0	4	2	0
40–<50	4	5	3	3	0	1	0
50–<60	5	2	7	5	0	0	1
60–<70	5	2	2	3	0	1	0
≥70	2	0	4	3	1	1	1
Pearson correlation coefficient (r)	0.055	–0.53	0.189	0.203	–0.112	0.039	#
P-value	0.586	0.125	0.058	0.041	0.263	0.695	#

Abbreviations: DIP – drug induced parkinsonism, NMS – neuroleptic malignant syndrome. * Others include chorea (n = 3), acute dyskinesia (n = 2), stereotypy (n = 3), myoclonic jerks (n = 1) and acute akathisia (n = 1). # Small sample size.

### 3.2. Phenomenology of DIMDs

**Figure 1:**
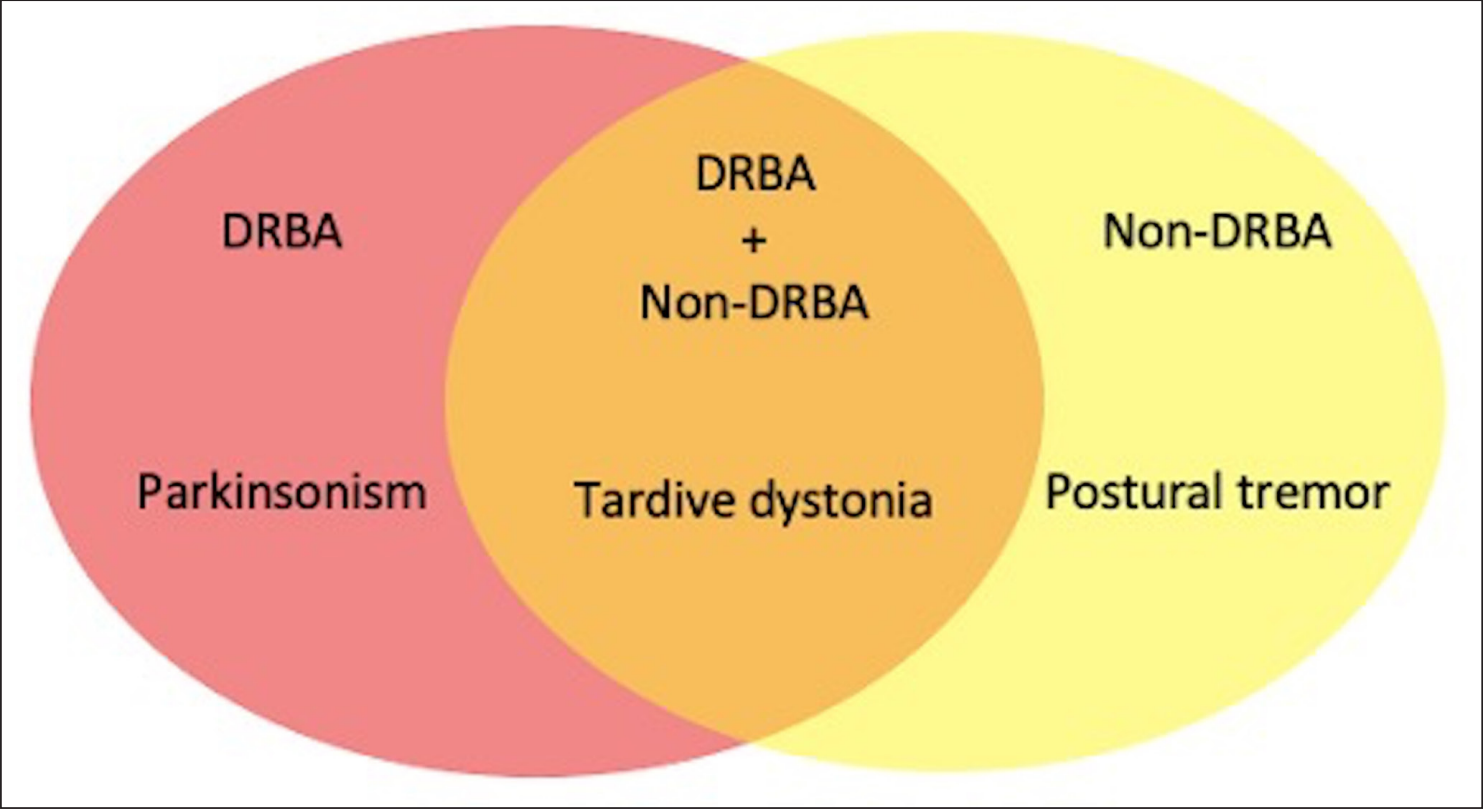
The most common movement disorders observed in different groups of drugs. DRBA: dopamine receptor blocking agents; Non-DRBA: Non-dopamine receptor blocking agents.

Different DIMDs observed in our 97 patients included tardive dystonia (n = 41; 42.2%), postural tremor (n = 38; 39.2%), parkinsonism (n = 32; 33%), tardive dyskinesia (n = 21; 21.6%), acute dystonia (n = 10; 10.3%), neuroleptic malignant syndrome (NMS) (n = 2; 2.1%), and others [(n = 10; 10.30%) including chorea and stereotypy each in 3; acute dyskinesia in 2; and myoclonic jerks and acute akathisia each in 1 patient] (See Video 1). Of 97 patients, 49 had more than one type of DIMDs, while 48 had a single type of DIMDs. Tardive dystonia was the most common DIMDs seen in DRBA+ non-DRBA group and the second most common in DRBA only and non-DRBA only group (Figure 1). The postural tremor was most common in the non-DRBA only group and the second most common in DRBA+ non-DRBA group. Drug-induced parkinsonism (DIP) was the most common DIMDs in DRBA only group.

**Videos:** **Phenomenology of drug induced movement disorders** Case 1. A 15-year-old boy developed parkinsonism after taking tablets of risperidone for 15 days. He also has truncal dystonia to the left and dystonic head tremor. Case 2. A 70-year-old female developed oro-lingual stereotyped tardive dyskinesia while on escitalopram for one year. There is also jaw dyskinesia in addition to lingual and labial dyskinesia. Case 3. A 12-year old girl developed acute jaw opening dystonia and oculogyric crisis after taking a single dose of tablet haloperidol (5mg). Case 4. A 65-year-old male developed stereotypy and respiratory dyskinesia while on tablet risperidone for 5 months. Case 5. A 43-year-old male who was a known case of hiatal hernia developed lingual dystonia while taking levosulpiride for one month for dyspepsia. Case 6. A 63-year-old male developed perioral and tongue dyskinesia, bruxism, and right upper and lower limbs stereotypy while on tablet haloperidol for 6 months for his psychiatric symptoms. Case 7. A 30-year-old man developed oculogyric crises on treatment with risperidone for 2 days. Case 8. A 60-year-old female developed acute onset akathisia after intramuscular injection of haloperidol (2 mg). There is also unusual posturing in the right hand.

### 3.3. Pharmacotherapy regimen characteristics of patients

Of these 97 patients, 30 (31%) had a history of DRBA intake, 37 (38%) had received non-DRBA, and 30 (31%) had received both DRBA and non-DRBA. The DRBA drug group included anti-psychotics and anti-emetics, while the non-DRBA drug group included anti-epileptics, anti-depressants, sedatives, anti-migraine drugs, mood stabilizer, and anti-cancer. The details of different DIMDs seen in 3 groups are presented in Table 1.

#### 3.3.1 DRBA group

The most common DIMDs seen in this group was DIP (53.3%), followed by tardive dystonia (36.6%). Out of 30 patients in this group, 21 patients had taken anti-psychotics, 8 patients had taken anti-emetics and 1 patient had received both anti-emetic and anti-psychotics.

#### 3.3.2 DRBA + non-DRBA group

The most common movement disorder seen in this group was tardive dystonia (56.7%) followed by postural tremors (12%). Out of 30 patients in this group, 26 had taken antipsychotics, 3 had anti-emetics and 1 had both antipsychotic and anti-emetic. Along with these DRBA, patients had taken non-DRBA which included anti-depressants in 12 patients, sedatives in 12 patients, anti-epileptics in 13 patients, mood-stabilizer in 3 patients and other drugs in 3 patients.

#### 3.3.3 Non-DRBA group

The most common DIMDs noticed in this group is the postural hand tremors (54%) followed by tardive dystonia (35.1%). Out of 37 patients in the non-DRBA group, the most commonly suspected drugs were anti-epileptics in 19 patients, followed by anti-depressants in 13 patients, then sedatives in 8 patients, calcium channel blockers (flunarizine) in 5 patients, other drugs like ranitidine, ciprofloxacin and vincristine each in one patient. It is important to note that calcium channel blockers (flunarizine) have more than one action on dopamine (pre and post synaptic).

The most common drug class implicated in causing DIMDs in our study was antipsychotics taken by 49 patients (including DRBA only and DRBA + non-DRBA group). The pharmacotherapy regimen characteristics of these patients are summarised in Table 3. Most of these patients (n = 31; 63%) received a single agent. Of 49 antipsychotic-induced movement disorder patients, 26 (53.1%) received second-generation antipsychotics (SGA), 9 (18.3%) received first-generation antipsychotics (FGA), and 14 (28.6%) received a combination of both SGA and FGA. Risperidone (n = 29, 59.2%) was the most common antipsychotic followed by haloperidol (n = 14, 28.6%).

**Table 3:** Pharmacotherapy regimen characteristics of patients who developed drug-induced movement disorders while taking anti-psychotics.

Characteristic	Description (N = 49)	

Agents^#^		
First generation agent (FGA):	Haloperidol	14 (28.6%)
	Trifluoperazine Chlorpromazine	5 (10.2%) 4 (8.2%)
	Fluphenazine	1 (2.04%)
Second generation agent (SGA):	Risperidone	29 (59.2%)
	Olanzapine	8 (16.3%)
	Aripiprazole	5 (10.2%)
	Amisulpride	3 (6.1%)
	Quetiapine	3 (6.1%)
	Clozapine	2 (4.1%)
Classification	FGA	9 (18.4%)
	SGA	26 (53.1%)
	FGA+SGA	14 (28.6%)
Total no. of agents	1	31 (63.3%)
	2	12 (24.5%)
	≥3	6 (12.2%)
Duration of exposure of neuroleptics before DIMD onset	<1 week	6 (12.2%)
	1 week–<1month	3 (6.1%)
	1month–<6month	10 (20.4%)
	6 months–<1year	4 (8.2%)
	1–<2 year	1 (2.04%)
	≥2 year	14 (28.6%)
	unknown	11 (22.4%)

# Total ≠ 100%, as some patients were taking multiple agents. Abbreviations: FGA – first-generation antipsychotics, SGA – second-generation antipsychotics, DIMD – drug-induced movement disorder.

We had a total of 13 patients who had a history of intake of the prokinetic drug. The details of these patients are summarised in Table 4. The most common prokinetic implicated was levosulpiride taken by 7 patients (6 females and one male). The mean ± [SD] age of the patients with levosulpiride induced movement disorder (LIMD) was 60.08 ± [9.20] years (range: 42–70 years). The mean [± SD] duration of levosulpiride intake before the onset of DIMDs was 13.85 [±16.83] months (ranging from one month to 4 years). Of these 7 patients, different types of movement disorders included DIP (n = 3), tardive dyskinesia (n = 4), postural tremor (n = 2), tardive dystonia (n = 2) and acute dystonia (n = 2).

**Table 4:** Details of patients with prokinetics induced movement disorders.

S No	Prokinetic agent	Sex	Age	Duration in months	DIP	Acute Dystonia	Tardive dyskinesia	Tardive dystonia	Postural tremor

1	Levosulpride	F	70	1 month	+	–	+	–	–
2	Levosulpride	F	70	2 months	+	+	–	–	–
3	Levosulpride	M	43	1 month	–	+	–	–	–
4	Levosulpride	F	42	4 years	–	–	+	–	+
5	Levosulpride	F	50	2.5 year	–	–	+	+	+
	Levosulpride	F	59	6 months	–	–	+	–	–
7	Levosulpride	F	60	9 months	+	–	–	+	–
8	Itopride	M	41	1 month	–	–	–	+	–
9	Itopride	F	38	15 days	+	+	–	–	–
10	Domperidone	F	26	1 week	–	+	–	–	–
11	Domperidone	M	22	1 month	–	–	–	+	+
12	Metoclopramide	M	55	8 months	–	–	+	–	+
13	Metoclopramide	M	76	3 months	–	–	+	–	–

Abbreviations: DIP – drug induced parkinsonism, NA – not available.

A history of intake of anti-epileptics was present in 31 DIMDs patients, out of which the majority (n = 17) had received only antiepileptics, while the remaining 14 patients had also received drugs of other groups like antipsychotics, anti-emetics, and anti-depressants. The details of the anti-epileptics induced movement disorder observed in our study are summarised in Table 5. Different DIMDs seen in these patients included postural tremor in 19 (61.3%), tardive dystonia in 11 (35.5%), DIP in 6 (19.3%), tardive dyskinesia in 4 (13%), and acute dyskinesia and chorea each in one (3%) patients.

**Table 5:** Pharmacotherapy regimen characteristics of patients with anti-epileptic induced movement disorders.

Characteristics	Detailed description (N = 31)	

Different subgroups of drugs taken	Only antiepileptic (without DRBAs) With DRBAs	17 (55%) 14 (45%)
Total number of antiepileptic agents	Monotherapy Polytherapy	24 (77%) 7 (22%)
Agents	Valproate	25 (80%)
	Phenytoin	7 (22%)
	Levetiracetam	4 (13%)
	Clobazam	2 (6%)
	Carbamazepine	1 (3%)
	Oxcarbamazepine	1 (3%)
Type of DIMD^@^	Postural tremor	19 (61.3%)
	Tardive dystonia	11 (35.5%)
	DIP	6 (19.3%)
	Tardive dyskinesia	4 (13%)
	Acute perioral tongue and palatal dyskinesia^#^	1 (3%)
	Chorea*	1 (3%)

@Total ≠ 100%, as some patients had more than one type of DIMD. # Phenytoin induced. * Valproate induced. DIP – drug induced parkinsonism, DRBA – dopamine receptor blocking agents, DIMD – drug-induced movement disorder.

### 3.4. Association of different DIMDs with different drug groups

The incidence of drug-induced parkinsonism (DIP) was significantly higher in DRBA only and DRBA + non-DRBA group as compared to non-DRBA only group (p-value = 0.0017). The non-DRBA and DRBA + non-DRBA group had a significantly higher incidence of postural hand tremor as compared to DRBA only group (p-value = 0.007).

## 4. Discussion

### 4.1. Epidemiology

We have described 97 DIMDs patients encountered in our tertiary care movement disorder center during the study period of 2 years. Most of our DIMDs patients belonged to the younger age group with the mean ± SD age at presentation being 35.89 ± 17.8 years (Range 2–80 years). Our observation differs from the published literature describing the higher incidence of DIMDs in the elderly population [9]. This difference of observations can be explained by the inclusion of a wide range of drugs including DRBA and non-DRBA causing DIMDs in our study, while most of the previous studies account for only DRBA. However, we noticed a significantly increased likelihood of tardive dyskinesia with age as observed in the previous literature [10]. The probable explanation for the effect of age is the decreased metabolic inactivation of drugs also leads to increased sensitivity to the drugs, particularly DRBA. We did not observe any significant gender bias in terms of different DIMDs. This is in contradiction with the previous studies demonstrating a higher incidence of neuroleptic-induced parkinsonism and tardive dyskinesia in females [11,12].

### 4.2. Different drugs causing DIMD

Previous studies have estimated the frequency of different DIMDs [9,10]. However, the frequency of different DIMDs varies in the different studies depending on the inclusion criteria. We observed a significant number of non-DRBA drugs causing DIMDs as compared to previous studies as the patients in our study were recruited from a movement disorder center while most of the previous studies on DIMDs are done by psychiatrists on the patients with underlying psychiatric illness who are on neuroleptics. Thus data from the previous studies do not represent the exact spectrum of the DIMDs patients encountered by neurologists. In our study, non-DRBA drugs were included only if they satisfied the criteria for probable or definite ADR in Naranjo scoring. The non-DRBA drug group included anti-epileptics (like valproate, phenytoin), anti-depressants (like TCA, SSRIs), mood stabilizers, (like lithium) and calcium channel blockers (like flunarizine) which have more than one action on dopamine (pre and post synaptic). These findings are interesting to consider in the context of existing literature describing tardive dyskinesia-like syndromes in patients who were never on DRBAs as an extraordinarily rare event and disapproving non-DRBAs like SSRIs, TCAs, as a causal agent until other explanations have been excluded [8]. In 31% of our DIMDs cases who took non-DRBA, there was a history of DRBA intake in the past at some point of time which might have caused some “priming” effect, which when followed by the addition of non-DRBAs, lead to the unmasking of “latent” tardive syndromes. In the patients who took both DRBA and non-DRBA drugs, the role of the non-DRBA drug in deciding the phenomenology is indicated by the differences in the relative frequency of different DIMDs in three subgroups. When comparing DRBA versus non-DRBA, the incidence of DIP was significantly higher in the DRBA group, and postural tremor was significantly higher in the non-DRBA group. But when patients took both DRBA and non-DRBA drugs, tardive dystonia was the most common DIMD. However, the exact mechanism is not known but some of these non-DRBA drugs produce DIMDs as a secondary effect on striatal dopamine levels through alteration of other neurotransmitters [13,14].

### 4.3. Dopamine receptor blocking agents (DRBA)

Among the DRBA group, DIP was the most commonly observed DIMDs. The pathophysiology of DIP caused by DRBAs is based on drug-induced changes in the basal ganglia motor circuit due to dopaminergic receptor blockade. Among DRBA, the anti-psychotics were the most common drug groups implicated in causing DIMDs. Out of the 49 DIMDs patients who received antipsychotics in our study, most of the patients (n = 26; 53.1%) received second-generation antipsychotics (SGAs), 9 (18.3%) received FGAs, and 14 (28.6%) received a combination of both SGAs and FGAs. Among antipsychotics, SGAs have been hypothesized to cause fewer extrapyramidal syndrome as compared to FGAs owing to their pharmacological properties like central serotonin-2A (5-HT2A) antagonism, fast D2 dissociation, and D2 partial agonism [15]. However, DIMDs are still common in patients on anti-psychotics, because there has been a dramatic increase in the prescription of SGAs.

Other DRBA drugs suspected for causing DIMDs in our study were prokinetic drugs taken by 13 (13.4%) DIMDs patients. The most commonly implicated drug among them was levosulpiride. The mean age of the patients with levosulpiride induced movement disorders (LIMs) was 60.08 ± [9.20] years (range: 42–70 years) and there was a high female preponderance (6/7; 85.71%), similar to previously reported in the literature [16]. DIP and tardive dyskinesia was present each in 4 out of 7 patients with LIMs. Interestingly one of these patients had co-existing DIP and tardive dyskinesia. The most prominent theories about DIP and tardive dyskinesia suggest that DIP results from the hypodopaminergic state because of dopaminergic receptor blockade while tardive dyskinesia results from the super-sensitivity of post-synaptic dopamine receptors due to chronic blockade, but this hypothesis is not supported by data. To explain this paradox of simultaneous presence of two conditions with opposite pathophysiologic mechanisms, Jankovic et al proposed regional striatal pharmacological differences because of the complex somatotopic organization of the basal ganglia [9,17]. Both the patients who took metoclopramide in our study had tardive dyskinesia. Other prokinetic agents included domperidone and itopride, which were supposed to be devoid of extrapyramidal side effects because of their low central nervous system penetration. There are few case reports of domperidone and itopride induced movement disorder in the literature [18,19,20,21].

Various medications including DRBAs and non-DRBAs like amiodarone, antidepressants, beta-adrenergic agonists, cyclosporine, lithium, and valproic acid, are known to induce non-parkinsonian tremors [22]. Different mechanisms proposed for drug-induced tremor include blockade of dopamine receptors in the basal ganglia (DRBAs), stimulation of serotonergic receptors found in the inferior olive (SSRIs) and enhancement of physiological tremor [14].

### 4.4. Non-dopamine receptor blocking agents

The postural tremor was the most common movement disorder seen in non-DRBA drug groups especially anti-epileptics, which is consistent with the finding of previous studies [23]. Out of a total of 97 DIMDs patients, 31 patients had a history of intake of anti-epileptics. The exact pathophysiology of anti-epileptic induced movement disorders is not known. In our study, valproate (n = 25, 80.6%) was found to be the most commonly taken antiepileptic drug and the most common movement associated was postural tremor (n = 17) followed by parkinsonism (n = 5). Valproate-induced movement disorders appear to be a dose-related side effect [24]. Phenytoin was the second most common antiepileptic (n = 7, 22.6%) drug to cause movement disorder in our study and the different DIMDs included postural tremor (n = 4), tardive dyskinesia (n = 3), tardive dystonia (n = 3), acute dystonia (n = 1) and acute dyskinesia (n = 1). Harrison et al. hypothesized the differential effect of phenytoin on dopamine receptor subtypes or their associated second messenger systems resulting in a disturbance in the functional equilibrium of the basal ganglia output systems leading to hyperkinetic movement [25].

Another subgroup of the drug observed to cause DIMDs in our study was flunarizine. We had 5 patients with a flunarizine-induced movement disorder in our study. Different movement disorders reported with flunarizine included parkinsonism, postural tremor, tardive dystonia, and chorea [28]. The exact pathological mechanism responsible for flunarizine induced parkinsonism is still not completely understood. However, it has been hypothesized that it is likely to be due to pre-synaptic (dopamine depletion due to loss of tyrosine hydroxylase in monoaminergic and serotonergic neurons) and post-synaptic (blocking dopaminergic receptors) factors [26,27].

### 4.5. Limitations

There are certain limitations to our study. First, our institute is a tertiary care centre located in north-India, and there was an inherent referral bias. Second, our center primarily caters to the adult population, so there is a limited representation of paediatric DIMDs in our study. Third, there is a lack of detailed follow-up data of the patients. Fourth, using the Naranjo scale with a cut-off of 5 is a relatively weak hurdle to overcome. Lastly, the sample size for individual drug subgroup is relatively small to conduct analyses for their association with individual DIMDs.

Despite these limitations, our study has provided robust data regarding the DIMDs in a movement disorder setting highlighting various DRBA and non-DRBA drugs causing DIMD. Prior studies have thoroughly investigated the role of DRBAs in DIMD [9]. However, little research has been conducted to identify the role of non-DRBA. Thus, the strength of our study lies in the broad spectrum of the reported phenomenology and the causative underlying drugs causing DIMDs encountered in the movement disorder setting.

## 5. Conclusion

Our study draws attention to the different types of DIMDs seen in DRBA and non-DRBA group of drugs, many of which are misdiagnosed. Our patients were younger than other studies reporting DIMDs. Tardive dystonia was the most common DIMDs observed in our study, particularly in DRBA + non-DRBA group. Among DRBAs, antipsychotics were most common with DIP being the most common DIMDs among them and a significant number of SGAs were implicated. Among non-DRBAs, anti-epileptics, especially valproate, were most frequently seen in association with DIMDs and postural tremor was the most common DIMDs among them. The early recognition and differentiation of DIMDs from other conditions have important pathophysiological and therapeutic implications.

## Abbreviations

1. DIMDs – Drug-induced movement disorders
2. DIP – Drug-induced parkinsonism
3. DRBA – Dopamine receptor blocker agents
4. DSM-5 – Diagnostic and Statistical Manual of Mental Disorders-5
5. EPS- Extrapyramidal symptom
6. FGA – First-generation antipsychotics
7. FIMD – Flunarizine induced movement disorder
8. GABA – Gamma amino butyric acid
9. LIM – Levosulpiride induced movement disorder
10. NMS – Neuroleptic malignant syndrome
11. SGA – Second-generation antipsychotics
12. SSRIs – Selective serotonin receptor inhibitors
13. TCA – Tricyclic antidepressants
